# Acute Effects of Beetroot Supplementation on Resistance Exercise Performance in Physically Active Men

**DOI:** 10.3390/sports14030094

**Published:** 2026-03-02

**Authors:** Maitê Yorioka Rodrigues, Monica Yuri Takito, Gabriel Albanese Kafouri, Rebeca Soares Pires, Felipe Gasperini Mello, Reza Zare, Sthefano Ventura Hernandez, Katie M. Heinrich, Emerson Franchini

**Affiliations:** 1 School of Physical Education and Sport, University of São Paulo, São Paulo 05508-030, Brazil; gabrielkafouri@usp.br (G.A.K.); felipe.gaspermello@gmail.com (F.G.M.); 2 Laboratory of Energetic Determinants of Sport Performance, School of Physical Education and Sport, University of São Paulo, São Paulo 05508-030, Brazil; 3 SRH Campus Hamburg, Berlin University of Applied Sciences, 20095 Hamburg, Germany; 4 School of Medicine and Health Sciences, Tecnológico de Monterrey Campus Guadalajara, Zapopan 45201, Mexico; 5 Department of Kinesiology, College of Health and Human Sciences, Kansas State University, Manhattan, KS 66506, USA; kmhphd@ksu.edu

**Keywords:** dietary nitrate, strength endurance, leg press, bench press

## Abstract

The objective of this study was to investigate the effect of nitrate (NO_3_^−^) supplementation on exercise performance in multiple sets of bench press (BP) and leg press (LP) at 80% of one-repetition maximum (1RM), to determine whether it would be beneficial towards the number of repetitions to failure (RTF). A total of 18 trained male subjects (25 ± 3 years old) completed two sessions of repeated number of maximum repetition (NMR) tests in BP and LP to assess RTF, power output, heart rate (HR), rating of perceived exertion (RPE) 2 h after NO_3_^−^ or placebo intake. Comparisons between dependent variables were conducted using a two-factor analysis of variance (ANOVA) with repeated measures, examining the factors of condition and sets. The results for RTF showed only a main effect of set for BP and LP. No significant differences were found between conditions for total RTF. Our results showed that the NO_3_^−^ supplementation had no significant effect on RTF, mean power, peak power, HR, and RPE when compared to placebo conditions. Results demonstrated that for physically active male individuals with experience in strength training, NO_3_^−^ supplementation did not affect strength endurance performance.

## 1. Introduction

Practitioners frequently use dietary supplements to enhance performance, particularly to increase total training volume by improving both repetition capacity and recovery between sets [1]. Over the past decades, researchers have investigated the ergogenic effects of dietary nitrate (NO_3_^−^)—commonly found in beetroot—across various exercise modalities, reporting positive outcomes in submaximal continuous efforts as well as high-intensity exercise protocols [2], including single- and multiple-bout strength exercises [3]. When training within specific intensity zones aimed at developing strength endurance, hypertrophy, or maximal strength, one widely recommended strategy involves the use of higher loads, typically around 80% of one-repetition maximum (1RM) [4,5,6]. However, despite this recommendation, there remains no scientific consensus regarding the most effective approach for optimizing these adaptations [7]. The expected mechanism underlying the effects of NO_3_^−^ involves its role as a precursor of nitric oxide (NO). Through the enterosalivary pathway, NO_3_^−^ is reduced to nitrite (NO_2_^−^) and subsequently to NO, increasing its bioavailability [8]. Elevated NO levels may influence oxygen consumption [9,10,11,12] and ATP expenditure [2,13], modulate excitation–contraction coupling [14,15], and promote vasodilation [16]. These physiological responses can enhance fatigue resistance during repeated muscle contractions [15,17], attenuate metabolic perturbations [2,18], and augment force production [19,20,21], thereby reducing the overall energetic cost of exercise. In recognition of this evidence, NO_3_^−^ is listed by the International Olympic Committee as an effective dietary supplement [1]. Given these properties, nitrate supplementation may provide acute performance benefits in strength training contexts for both recreational practitioners and athletes. Empirical evidence supports these potential benefits. For example, studies by Coggan et al. [19,20] demonstrated that acute NO_3_^−^ supplementation increased peak power during knee extension in untrained men and women (*p* < 0.05), with a significant correlation between peak power and plasma NO_2_^−^ concentration (r = 0.60; *p* < 0.01) [19]. However, no improvements were observed in muscle function during 50 consecutive knee extensions [20]. Complementing these findings, a recent systematic review [22] reported that NO_3_^−^ supplementation significantly increased the number of repetitions to failure (RTF) compared with placebo, both in bench press (BP) and back squat exercises. In addition, significant improvements were observed in peak power and mean power during back squat performance [22]. Despite these promising findings, the current evidence remains limited and inconclusive, particularly regarding high-intensity, multi-joint strength-endurance exercises [22]. This lack of consensus is reflected in the heterogeneity of results observed across acute supplementation protocols, which typically range from ~300 to 800 mg of NO_3_^−^. Results have been demonstrated for a spectrum of dosage administration, mostly for 400 mg and 800 mg [22]. Importantly, current data do not support a clear dose–response relationship for strength performance, suggesting that increasing nitrate intake does not necessarily enhance neuromuscular outcomes [23]. Even though the focus of the present study was the acute effects of NO_3_^−^ supplementation, studies indicated that chronic doses have the potential to increase exercise tolerance and resistance training performance [24,25]. Moreover, inter-individual variability further complicates interpretation, as responsiveness to nitrate supplementation appears to be influenced by baseline NO status, oral microbiome composition, habitual dietary nitrate intake, and training background [3,26]. Additionally, positive effects are more frequently observed in isolated or single-joint exercise models, whereas findings in multi-joint, high-load exercises such as the BP and back squat are less consistent [14,19,20]. Thus, the primary aim of this study was to investigate the effect of NO_3_^−^ supplementation on performance in multiple sets of BP and leg press (LP) at 80% of 1RM. The hypothesis was that NO_3_^−^ supplementation is beneficial in maintaining performance and increasing the RTF throughout the sets, specifically, in the last sets of the strength exercises.

## 2. Materials and Methods

A total of 19 males (mean ± SD: 86 ± 17 kg; 179 ± 7 cm; 25 ± 3 years old) with experience in strength training (5 ± 2 years) were recruited via posters, social media, and institutional university email. All tests were performed after the consent form was explained and signed. Participants were eligible for inclusion if they met the following criteria: (a) males aged between 18 and 35 years old; (b) a minimum of 6 months of strength training experience; (c) free from neuromuscular problems. Participants were excluded if they were unable to execute the tests in the allotted time or made use of illegal performance-enhancement drugs (e.g., anabolic steroids).

**Figure 1:**
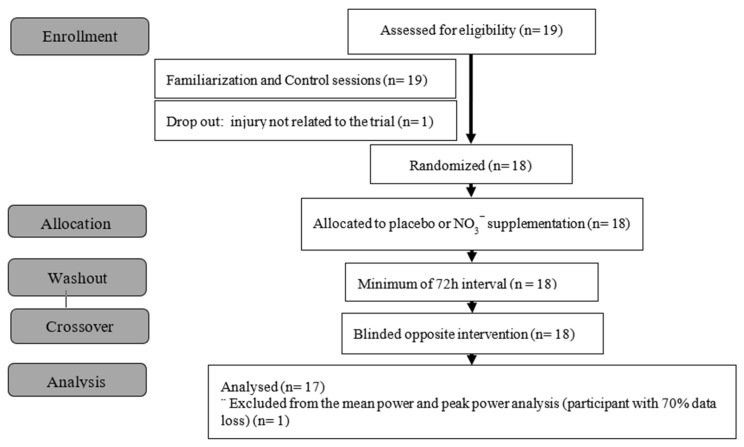
CONSORT flow chart.

The sample size was calculated using four factors: (a) number of maximum repetitions at 80% of 1RM, 9 ± 2 repetitions for BP and 13 ± 4 repetitions for LP [27]; (b) in a study, Panissa et al. [28], observed a drop of 30%, 40% and 40% of repetitions for the second, third and fourth sets of a half squat exercise in relation to the first set, with half squat exercise, and, therefore, the total number of repetitions was 56 ± for LP and 34 ± for BP; (c) the mean difference between NO_3_^−^ supplementation and placebo reported in the last set (mean difference 3.1 ± 4.8 repetitions) in a study by Williams et al. [29], with 3 sets at 70% of 1RM; and (d) the total number of repetitions in each exercise was considered the dependent variable, with 80% power and alpha *p* < 0.05. This calculation indicated the necessity of 17 participants. Even though 19 participants were recruited, only 18 remained until the last tests (one participant dropped out for personal reasons unrelated to the study) (Figure 1).

**Figure 2:**
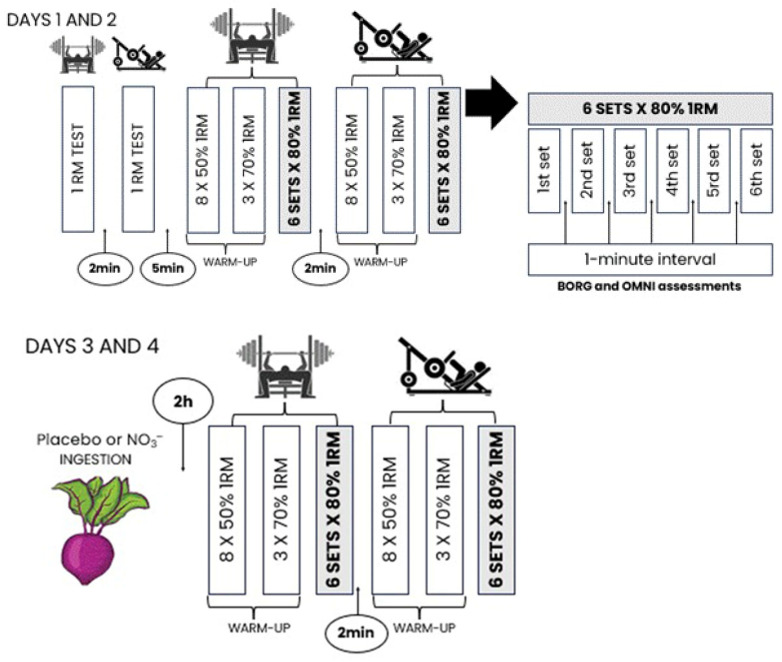
Experimental design.

The present study was conducted as a double-blind, crossover, randomized, placebo-controlled trial. The participants first completed a familiarization session, which consisted of a 1RM test in BP and LP, followed by repeated maximum repetition tests (NMR) for both exercises (Figure 2). All participants and assessors were blinded throughout data collection and processing. In a subsequent session, designated as a control session, on a different day, participants underwent the same procedures as the familiarization. After randomization, participants were invited to engage in two additional sessions, one preceded by the ingestion of NO_3_^−^ (beetroot powder) and another preceded by the ingestion of a placebo. All sessions were conducted with a minimum of 72 h washout period between them. The subjective measurements of perceived exertion (Borg and OMNI), Perceived Recovery Status (PRS), and heart rate (HR) were assessed throughout all sessions.

The 1RM test began with a general warm-up, consisting of 5 min on an ergometric bicycle, followed by 2 sets of BP or LP. In the first set, participants performed 8 repetitions at 50% of 1RM (estimated). For the second set, they did 3 repetitions at 70% of 1RM (estimated), with a 2 min interval between sets. After the specific warm-up, participants rested for 2 min and then performed up to 5 tries, with 3 to 5 min of rest between tries to establish their 1RM value [28]. This value represented the greatest mass lifted in each exercise with proper execution, as indicated by De Souza, Tricoli, and Franchini [30].

For the NMR test, participants started with a general warm-up, identical to the description above for the 1RM test. They then executed 6 sets of each exercise at 80% of 1RM, with 1 min rest intervals between sets and 2 min rest between exercises. The 1 min break was adopted in accordance with Schoenfeld et al.’s [7] work challenging common practices for strength training and Salles et al.’s [31] work on rest intervals for strength endurance. While Schoenfeld et al. [7] discuss the lack of strong evidence for the typical recommendations within the repetition continuum, Salles et al. [31] emphasize how shorter rest intervals benefit localized muscular and aerobic endurance (resulting in greater oxygen consumption), and that “extremely short intervals may allow for greater maintenance of relative force levels” (pg. 775). Suffice to say that for the proper maintenance of repetitions, longer rest intervals would be advised; however, our objective was to elicit performance decrements so that the effects of nitrate supplementation would be evident. Execution velocity was self-selected, similar to normal training sessions and in other studies [28,30,32]. At the end of each set, subjective assessments were taken (Borg and OMNI). During all sets, velocity was monitored by a linear encoder (Vitruve encoder; Madrid, Spain), to enable calculation of mean power and peak power completed in each experimental session. To ensure participants were in similar conditions before each experimental session, a recovery scale (PRS) was used. To secure proper technique and a full range of motion in the BP exercise, all repetitions were visually controlled, accounting for full elbow extension at the start of each repetition and the lowering of the bar to the chest. The repetition was completed when the elbows returned to full extension. For the LP exercise, participants were positioned on the machine with both feet on the platform, and after unlocking the machine, they lowered the platform until their knees reached a 90-degree angle. The repetition was completed when the knees returned to full extension. The position of the platform was measured using a tape beside the machine’s track, and a plastic marker was placed to indicate the 90-degree angle, ensuring proper form for each repetition.

The NO_3_^−^ supplementation followed procedures by Williams et al. [29]: 20 g of beetroot powder (Dobro BT 400 NIT, Dobro, São Paulo, Brazil) mixed with 100 mL of tea (Orange and strawberry caffeine-free iced tea, Leão, São Paulo, Brazil) or placebo (Orange and strawberry caffeine-free iced tea, Leão, São Paulo, Brazil) 2 h before each exercise session. The beetroot powder was standardized to provide approximately 400 mg of NO_3_^−^ per dose (20 g of powder), while the placebo provided none to insignificant amounts of NO_3_^−^. The dosage was selected based on the prior literature, specifically the systematic review and meta-analysis by Tan et al. [22], in which 3 of 6 studies using this dosage reported significant effects on RTF for either BP or back squat. Participants were advised to consume the totality of the liquid and to plug their noses while doing so, and ingestion was administered through a black squeeze bottle. Dietary instructions were to consume the same foods and beverages on the day of the experimental sessions. Drinks were distributed to all participants by a third party, not involved in the testing, and the order of distribution was only revealed to the researchers after the completion of the data collection.

A list of food sources that can contain a considerable amount of NO_3_^−^ was given to the participants, with instructions to avoid them 24 h prior to their arrival at the laboratory (beetroot, celery, arugula, lettuce, spinach, turnip, endive, parsley, cabbage, leek, etc.). Beverages containing caffeine, as well as fruits and vegetables rich in NO_3_^−^, were forbidden 72 h before tests due to their ergogenic potential. On the day of the tests, water was provided ad libitum during the rest periods between tests.

The HR was assessed using a sensor (Polar sensor H10; Polar Electro Brazil Comercio, Cajamar, Almería, Spain) attached to the subject’s chest. Immediately after each set, HR information was registered in a data collection sheet.

The Borg scale of perceived exertion [33], the OMNI picture system of perceived exertion [34], and the PRS [35] were used to extract subjective assessments between sets (Borg and OMNI) and between sessions (PRS). The rate of perceived exertion (RPE) was recorded after each set during the 1 min interval of the NMR test. Participants were presented with two separate sheets displaying the Borg and OMNI scales and asked to report the number corresponding to their perceived exertion. The perceived recovery was assessed at the beginning of each session, starting from session one, in the same way as the perceived exertion. The three scales were previously explained to each subject, and all information was registered in a data collection sheet.

Data were reported as mean ± standard deviation. Comparisons between dependent variables were conducted using a two-factor analysis of variance (ANOVA) with repeated measures, examining the factors of condition and sets. Mauchly’s test of sphericity was applied, and when assumptions were violated, Greenhouse–Geisser corrections were implemented to meet the requirements of repeated measures ANOVA. Post hoc analyses were conducted using the Bonferroni adjustment. The effect size was calculated using eta partial squared, and interpretation was made using Cohen’s proposal [36]. The initial data analysis was performed by a professor who was blinded to the group allocation to minimize potential bias and ensure objectivity in the evaluation of the results.

Considering the data from the familiarization and from the control condition, the intraclass correlation coefficient (ICC), typical error (TE), coefficient of variation (CV), and smallest worthwhile change (SWC) were calculated to determine reliability, and the 95% confidence interval (95%CI) was also calculated. For the NMR test, reliability was conducted like the 1RM tests, but we also calculated the SWC for various effect sizes (0.2, 0.6, and 1.2) by multiplying these values by the between-subject standard deviation (SD), following Cohen’s principle. The usefulness of each variable in both studies was then evaluated by comparing the SWC score to the typical error (TE) [37]. When the TE exceeded the SWC, the variable’s evaluation was classified as “marginal”. If the TE was comparable to the SWC, it was rated as “medium”, whereas a TE smaller than the SWC resulted in a “good” evaluation for detecting small (0.2), medium (0.6), and large (1.2) differences. Additionally, the MDC (minimum detectable change) was calculated using the formula TE × 1.96 × √2 and interpreted as the smallest change in a variable that allows us to confidently identify a true change [38].

## 3. Results

### 3.1. Reliability Analysis

In the NMR test, in BP, the following values were found (mean value and 95%CI): ICC = 0.890 (0.697, 0.960); TE = 3.0 (1.6, 4.4) reps; and CV = 15.97 (8.37, 23.55) %. The SWC was 1.3 (0.7, 2.0), 4.0 (2.1, 5.9), and 7.9 (4.2, 11.7) reps for 0.2, 0.6, and 1.2, respectively, presenting a good probability for detecting medium changes. The MDC was 8.3 (4.3, 12.2) reps. For the LP, these values were: ICC = 0.686 (0.134, 0.886); TE = 6.5 (3.4, 9.6) reps; and CV = 21.3 (11.18, 31.43) %. The SWC was 1.9 (1.0, 2.7), 5.6 (2.9, 8.2), and 11.1 (5.8, 31.4) reps, for 0.2, 0.6, and 1.2, respectively, presenting a good probability for detecting close to medium changes. The MDC was 18.0 (9.4, 26.5) reps. For the 1RM test, in BP, the following values were found (mean value and 95%CI): ICC = 0.990 (0.972, 0.996); TE = 3.3 (1.7, 4.8) kg; and CV = 3.52 (1.85, 5.19)%. For the LP, these values were: ICC = 0.978 (0.940, 0.992); TE = 17.8 (9.4, 26.3) kg; and CV = 4.54 (2.38, 6.70)%.

At the end of the testing period, participants were asked to identify which session they believed they had consumed the supplement or the placebo, and 50.0% correctly identified what they had ingested, which is exactly the proportion expected in a guess based purely on chance (50%).

### 3.2. Data Loss

During the tests, ongoing data checks revealed an 8.1% data loss from the encoder (for total RTF in control, placebo, and supplemented conditions, with 90.2% of this problem concentrated in the leg press exercise, and a similar loss for each condition. This affected only the mean power and peak power variables, due to the encoder’s incapability to detect movements under 0.04 m/s, especially in the last repetitions (1.4%) and due to errors in device manipulation (6.7%). One subject was removed from the mean power and peak power analysis because of a 71% loss in data from the leg press exercise (representing 36% of all data loss) (Figure 1). It is important to point out that a few participants in different sessions, for the last four sets, could not execute a single repetition (failure to complete one repetition in the set; in these cases, zero was inserted), and that was accounted for in the means and SDs for all variables. Repeating testing sessions in which greater data loss occurred would be advised to minimize data loss, as well as making use of two encoders simultaneously.

### 3.3. Heart Rate

For the HR responses (BP: control = 125 ± 12 bpm; placebo = 126 ± 15 bpm; NO_3_^−^ = 124 ± 15 bpm; LP: control = 141 ± 15 bpm; placebo = 144 ± 18 bpm; and NO_3_^−^ = 143 ± 16 bpm) to the BP and LP exercises, no statistically significant effects or interactions were observed (*p* > 0.096).

### 3.4. Borg’s RPE and OMNI Responses

**Figure 3:**
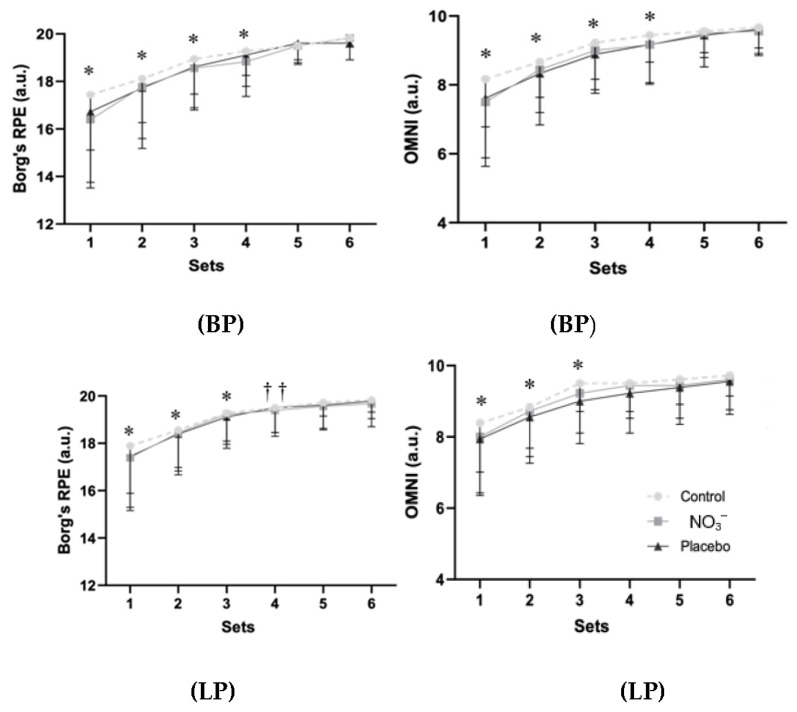
Ratings of perceived (RPE) exertion responses using Borg 6–20 and OMNI scales during six sets of bench press (BP) and leg press (LP) exercises at 80% of 1RM in the control, placebo, or nitrate supplementation (NO_3_^−^) conditions. Data are presented as mean ± SD. *, significantly lower than all subsequent sets (*p* < 0.05). ††, significantly lower than the last set (*p* < 0.01).

Borg’s RPE (F_1.48,75.69_ = 56.58, *p* < 0.001, η_p_^2^ = 0.526, large) and OMNI (F_1.67,85.47_ = 58.71, *p* < 0.001, η_p_^2^ = 0.535, large) responses significantly differed across the sets (Figure 3) for BP and for LP (Borg’s: F_1.46,74.59_ = 54.36, *p* < 0.001, η_p_^2^ = 0.516, large; OMNI: F_1.60,81.73_ = 45.99, *p* < 0.001, η_p_^2^ = 0.474, large).

### 3.5. Power Output

**Figure 4:**
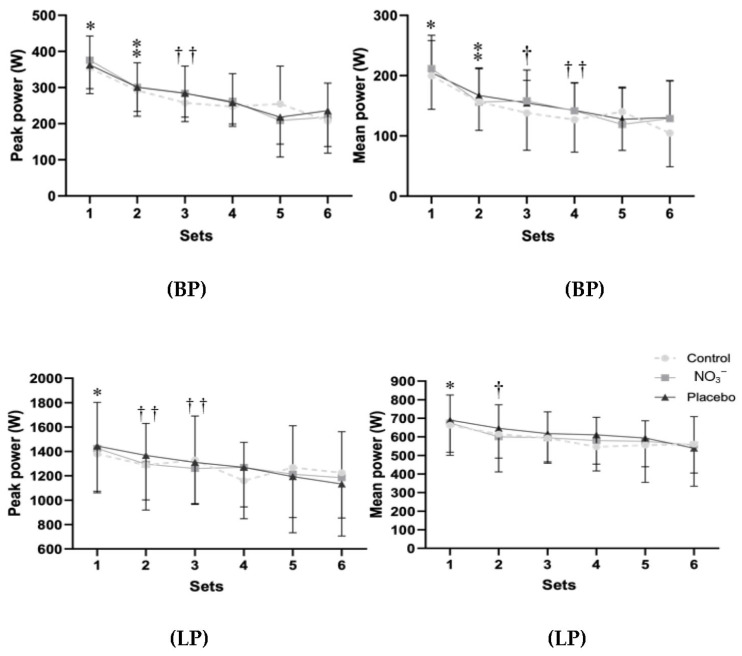
Peak and mean power in each of the six sets of bench press (BP) and leg press (LP) exercises at 80% of 1RM in the control, placebo, or nitrate supplementation (NO_3_^−^) conditions. Data are presented as mean ± SD. *, significantly higher than all subsequent sets (*p* < 0.004). ⁑, significantly higher than the last 3 sets (*p* < 0.003). †, significantly higher than the last 2 sets (*p* < 0.05). ††, significantly higher than the last set (*p* < 0.05).

**Figure 5:**
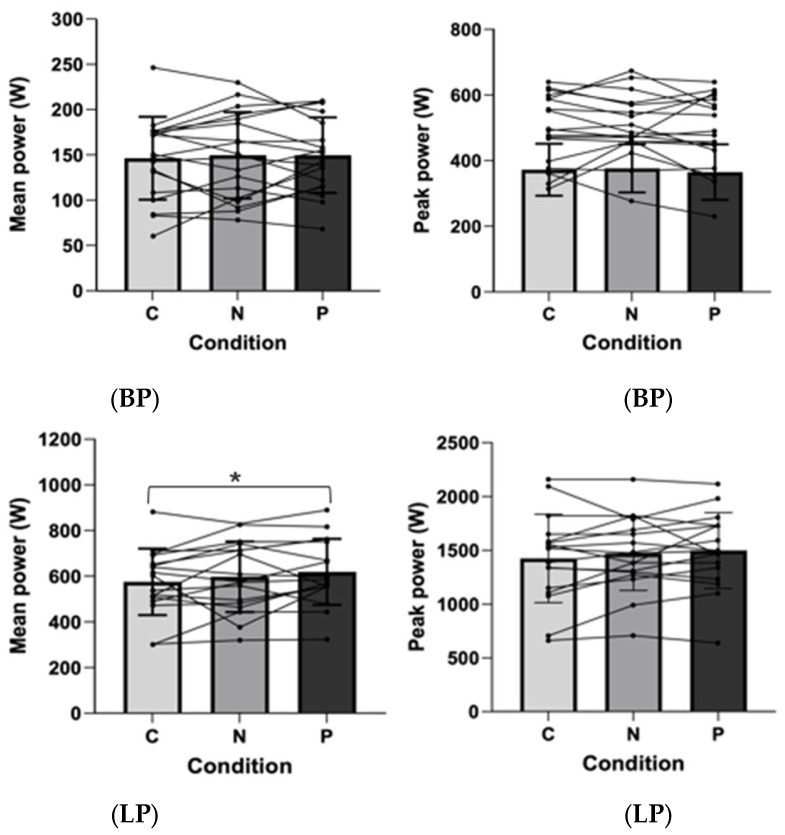
Mean power and peak power for the control (C), nitrate (N), and placebo (P) conditions in the bench press (BP) and leg press (LP) exercises at 80% of 1RM. Mean power for LP in the placebo condition was significantly higher than control (*p* < 0.05); For all others, no significant difference was found (*p* > 0.05). Data are presented as mean ± SD. *, significantly higher for the placebo condition compared with the control condition (*p* < 0.05).

Results indicated a significant main effect of set in the BP exercise (Figure 4), for peak power (F_3.35,160.69_ = 37.53, *p* < 0.001, η_p_^2^ = 0.439, large) and for mean power (F_3.20,153.62_ = 39.98, *p* < 0.001, η_p_^2^ = 0.454, large), as well as for the LP exercise’s peak power (F_3.22,138.67_ = 11.31, *p* < 0.001, η_p_^2^ = 0.208, large) and mean power (F_3.25,139.64_ = 13.33, *p* < 0.001, η_p_^2^ = 0.237, large). Figure 5 shows the average mean power and peak power in the BP and LP exercises for each condition. No significant differences were found between conditions (*p* > 0.05), except for the mean power in LP, which showed significantly higher values for the placebo condition when compared to the control condition (*p* < 0.02).

### 3.6. Repetitions to Failure

**Figure 6:**
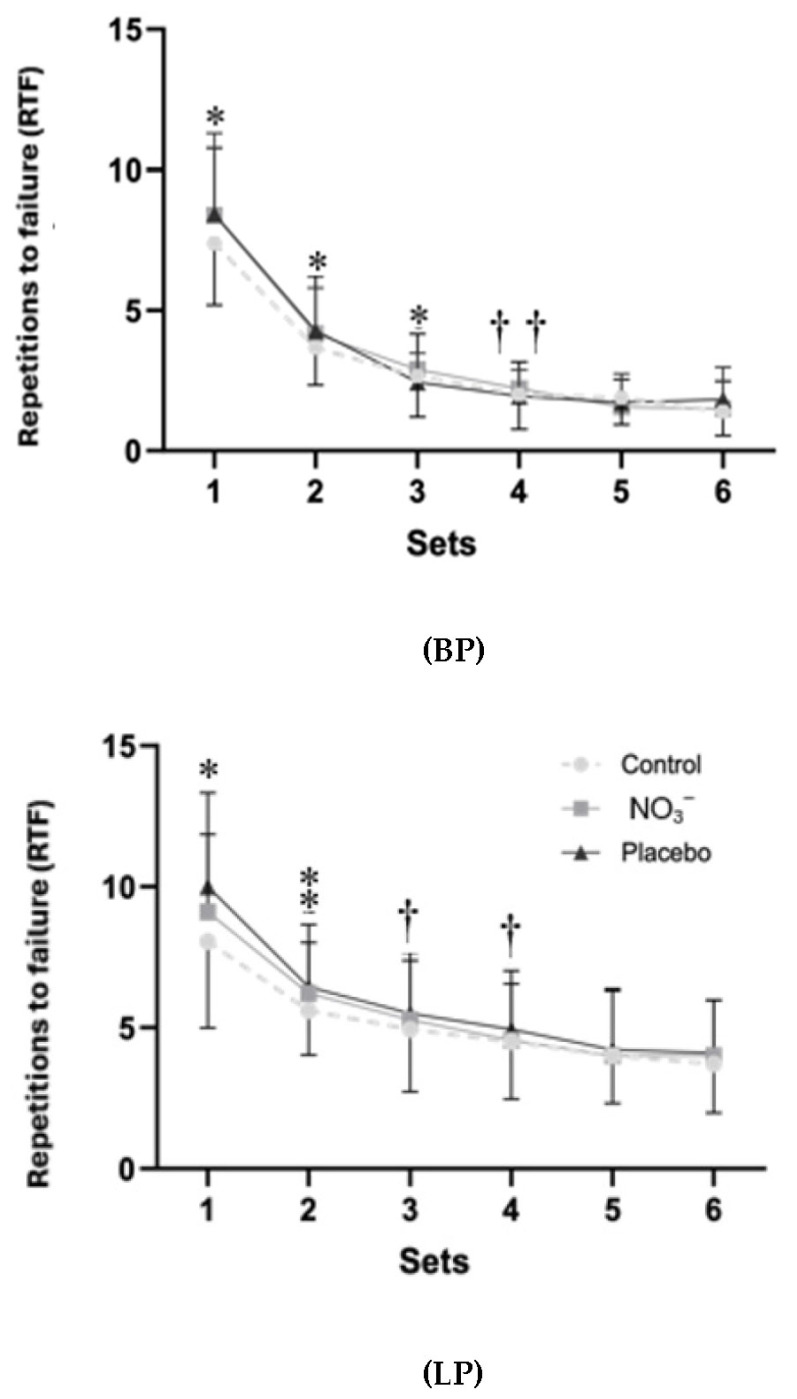
Repetitions to failure (RTF) in each of the six sets of bench press (BP) and leg press (LP) exercises at 80% of 1RM in control, placebo, and nitrate supplementation (NO_3_^−^) conditions. Data are presented as mean ± SD. *, significantly higher than all subsequent sets (*p* < 0.001). ⁑, significantly higher than the last 3 sets (*p* < 0.001). †, significantly higher than the last 2 sets (*p* < 0.001). ††, significantly higher than the last set (*p* < 0.001).

**Figure 7:**
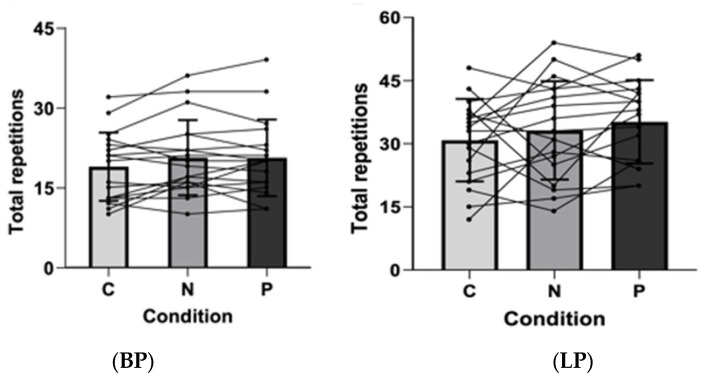
Total repetitions to failure (RTF) for bench press (BP) and leg press (LP) in control (C), nitrate (N), and placebo (P) supplementation conditions at 80% of 1RM. No significant differences were found between conditions for total RTF (*p* > 0.05). Data are presented as mean ± SD.

The results for RTF (Figure 6) showed only a main effect of set for BP (F_2.32,118.34_ = 287.68, *p* < 0.001, η_p_^2^ = 0.849, large) and LP (F_2.32,115.25_ = 86.95, *p* < 0.001, η_p_^2^ = 0.630, large). Figure 7 shows the total number of RTF for the BP and LP exercises for each condition. No significant differences were found between conditions for total RTF (*p* > 0.05).

## 4. Discussion

This study aimed to investigate whether the intake of 400 mg of NO_3_^−^ supplementation could enhance strength-endurance performance during six sets of BP and LP at 80% of 1RM with one-minute rest intervals between sets. Contrary to our hypothesis, our results showed that supplementation did not enhance performance in terms of total RTF and power output. All variables presented a similar behavior between conditions throughout the sets, increasing (RPE) or decreasing (RTF, mean power, and peak power) from first to last set, apart from HR, suggesting that the cardiovascular demand was similar for each set, considering the one-minute interval rest period [39,40,41].

The NO_3_^−^ supplementation effect on strength endurance relies on the premise that as energy storage gets lower, mostly from phosphocreatine (PCr) usage, the higher oxygen availability, provided by the nitric oxide production pathway, would facilitate PCr resynthesis throughout the six sets. Additionally, it could impact ATP turnover [2] and oxygen consumption efficiency [9,10,11], enhancing the possibility of increased RTF as compared to placebo. One possible explanation for the lack of significant effects in our study is that the one-minute rest interval may have been insufficient to fully resynthesize phosphocreatine (PCr), despite the potential for enhanced oxygen availability from the NO_3_^−^ supplementation [42]. At higher intensities, muscle recruitment increases along with substrate depletion as a result of intense muscle contraction and accumulated fatigue, negatively impacting exercise performance. This impact could be attenuated, specifically related to subsequent bouts of maximal efforts, when preceded by rest intervals where PCr resynthesis allows recovery, given that PCr levels could be significantly reestablished between the first and the second minute of recovery [43]. Therefore, higher intensities can induce a greater disturbance in homeostasis, meaning the larger oxygen availability, induced by NO_3_^−^ supplementation, could be redirected to reestablishing basal levels, possibly having no impact on between-sets recovery. The decrease in PCr concentration, significantly diminishing the muscle’s capability to maintain performance, combined with increased RPE, could explain the behavior of the RTF, mean power, and peak power throughout the six sets, especially when comparing the first sets with the last ones.

The unique characteristics of our study, specifically the intensity (80% of 1RM) and volume (six sets), combined with the limited number of studies examining resistance exercise and NO_3_^−^ supplementation, create challenges in comparing results across different research studies. Nevertheless, certain similarities allow for the formulation of inferences. The possible effect of NO on muscle force production was demonstrated by Bailey et al. [2] at high intensity work rates (30% maximum voluntary contraction) after 6 days of supplementation, by Whitfield et al. [21] and Haider and Folland [14] associated with low frequency stimulation after 7 days of supplementation, all on single joint exercises, involving chronic intervention and/or involuntary contraction. Focusing on power output, Tan et al. [17] observed no effect of 800 mg acute NO_3_^−^ supplementation during a single set of BP and back squat exercises at 60% of 1RM. However, Tan et al. did report a significant difference in the RTF for the BP between placebo and supplemented conditions, a finding not observed in our study during the initial sets. The aforementioned study’s results are consistent with those of two other studies, concerning the RTF in BP, which employed 70% [29] and 60% of 1RM [24], both with 2 min rest intervals. In contrast, Ranchal-Sanchez et al. [44] found no effects of 400 mg acute NO_3_^−^ supplementation on RTF or power output in an incremental test for BP at 60%, 70%, and 80% of 1RM with three-minute rest intervals. In this study, the back squat performance showed increased RTF under the supplemented condition at 60% and 70% of 1RM. The discrepancies between our findings and those of these studies may be attributed to differences in exercise intensity (60% and 70% of 1RM), rest intervals (2–3 min), and the dosage of supplementation used (up to 800 mg of NO_3_^−^).

Our finding provides an important contribution to the current literature by helping to delineate the contexts in which NO_3_^−^ supplementation is effective and where it may not be beneficial. Such evidence is critical for practitioners, coaches, and athletes when making evidence-based decisions about supplementation strategies. Based on this section’s previous observation, we suggest that future research should consider longer rest intervals, particularly at higher intensities, to better assess the effects of NO_3_^−^ supplementation, within this protocol. Additionally, it would be advised to test strength endurance aligned with high-intensity strength training protocols to better assess the supplementation effect in situations closer to practitioners’ reality. Based on the current evidence, NO_3_^−^ supplementation may be most relevant for strength-training contexts that prioritize fatigue resistance, power maintenance, or repeated submaximal efforts, particularly in single-joint or controlled exercise modalities [2,14,19,20]. Practitioners should be cautious when applying NO_3_^−^ supplementation to high-intensity, multi-joint strength training performed at ≥80% of 1RM with short inter-set rest intervals, as performance benefits under these conditions are inconsistent. Additionally, increasing NO_3_^−^ dosage does not appear to guarantee enhanced strength outcomes [23], highlighting the importance of individualized approaches that consider exercise selection, training intensity, recovery duration, and potential inter-individual responsiveness when integrating NO_3_^−^ supplementation into training programs [45].

Although this was a randomized, double-blind, and placebo-controlled trial with blinding at multiple levels, from supplement administration to statistical analysis, some limitations were present. First, there was the impossibility of determining the optimal individual dose–response of the NO_3_^−^ for each. Second, no control was implemented to prevent participants from self-pacing across the six sets, which may have led some participants to conserve effort to complete all sets. However, this behavior was not clearly detected during the investigation. Third, the control condition was not included in the randomization to establish some stability in the RTF throughout the six sets for the other two conditions. Fourth, given that the sample size of the present study did not allow the division of participants based on maximal strength performance, it is recommended that future studies address whether NO_3_^−^ ingestion could affect individuals with different strength training statuses. Finally, exercise order was not randomized, which may have influenced performance outcomes.

## 5. Conclusions

Our results demonstrated that for physically active individuals with experience in strength training, 400 mg of NO_3_^−^ supplementation did not significantly affect strength endurance after 2 h of intake in BP and LP exercises.

## Data Availability

The original data presented in the study are openly available in FigShare at https://doi.org/10.6084/m9.figshare.30411049.

## Abbreviations

The following abbreviations are used in this manuscript:

BP	Bench press
LP	Leg press
1RM	One-repetition maximum
RTFs	Repetitions to failure
NMR	Number of maximum repetitions
HR	Heart rate
RPE	Rate of perceived exertion
PRS	Perceived recovery status
ICC	Intraclass correlation coefficient
TE	Typical error
CV	Coefficient of variance
CI	Confidence interval
SWC	Smallest worthwhile change
MDC	Minimum detectable change
PCr	Phosphocreatine
